# Supplementary material for: Psychometric properties of the Multi-group Ethnic Identity Measure (MEIM) in a sample of Iranian young adults
Source: PeerJ. 2021 Feb 23;9:e10752. doi: 10.7717/peerj.10752 (PMC7908888; doi:10.7717/peerj.10752)
Supplement: Supplemental Information 3 [file peerj-09-10752-s003.pdf]

	این سوالات مربوط به قومیت یا گروه قومی شما و نحوه برخورد شما با آن یا واکنش شما نسبت به آن است. ازگزینه های زیر استفاده کنید تا نشان دهید که با هر یک از این عبارات چقدر موافق یا مخالف هستید.	کاملا موافقم	موافقم	نظری ندارم	مخالفم	کاملا مخالفم
۱	زمان زیادی را صرف تلاش برای یافتن اطلاعات بیشتر راجع به گروه قومی خود، مانند تاریخ و آداب و رسوم آن کرده‌ام.					
۲	در گروه‌های اجتماعی یا تشکل هایی فعال هستم، که بیشتر اعضای آن از قومیت من هستند.					
۳	درک روشنی از تاریخچه قومی خود و اینکه چه معنایی برایم می‌دهد، دارم.					
۴	در مورد اینکه چگونه در آینده زندگی من می تواند تحت تاثیر قومیت من قرار گیرد، زیاد فکر می کنم.					
۵	از اینکه عضو این گروه قومی هستم، خوشحالم.					
۶	وابستگی زیادی نسبت به گروه قومی خود احساس می‌کنم.					
۷	به خوبی درک می‌کنم که عضویت در گروه قومی‌ام برای من چه معنایی دارد.					
۸	برای آنکه درباره تاریخ قومی خود بیشتر بدانم، غالباً با دیگران راجع به فرهنگ خود گفت وگو می‌کنم.					
۹	به گروه قومی خود افتخار می‌کنم.					
۱۰	در آداب و رسوم فرهنگی گروه خود مانند غذاهای مخصوص، موسیقی یا رسم و رسومات، مشارکت می-کنم.					
۱۱	دلبستگی عمیقی نسبت به گروه قومی خود احساس می‌کنم.					
۱۲	در خصوص تاریخ فرهنگی یا قومی خود احساس خوبی دارم.					
۱۳	قومیت من:<br>(۱) آسیایی یا آسیایی آمریکایی، شامل چینی، ژاپنی و سایر. ☐<br>(۲) سیاهپوست یا آفریقایی آمریکایی. ☐<br>(۳) اسپانیایی یا لاتین، شامل مکزیکی آمریکایی، آمریکای مرکزی، و سایر. ☐<br>(۴) سفیدپوست، قفقازی، انگلو (نژاد انگلیسی)، اروپایی آمریکایی؛ اسپانیایی نیستم. ☐<br>(۵) آمریکایی هندی/ بومیان آمریکایی. ☐<br>(۶) ترکیبی؛ والدین از دو گروه متفاوت هستند. ☐<br>(۷) سایر اقوام (بنویسید): --					
